# Robson 10-groups classification system to access C-section in two public hospitals of the Federal District/Brazil

**DOI:** 10.1371/journal.pone.0192997

**Published:** 2018-02-20

**Authors:** Cláudia Vicari Bolognani, Lílian Barros de Sousa Moreira Reis, Adriano Dias, Iracema de Mattos Paranhos Calderon

**Affiliations:** 1 Medical School Coordination, Graduate School of Health Sciences/FEPECS/SES, Brasília, Federal District, Brazil; 2 Graduate Program in Gynecology, Obstetrics and Mastology, Botucatu Medical School/UNESP, Botucatu, São Paulo, Brazil; National Yang-Ming University, TAIWAN

## Abstract

**Background:**

The global increase in C-section rates is real. In Brazil, these indices correspond to 58.94% in the Midwest region and 52.77% in the Federal District.

**Objective:**

To evaluate the C-section rates and identify the groups with the greatest risk at two reference hospitals in the public network of Federal District/Brazil, using 10-Group Robson System.

**Method:**

A cross-sectional study of 6579 births assisted at the Hospital A (HA) and the Hospital B (HB) during 2013. The C-section rates in each group and its respective contribution to the total hospital C-sections was compared between HA and HB. To this, was used the proportion difference test (similar to chi-square test), with RR and 95% CI, and the logistic regression analysis (OR; 95% CI) among the groups with higher C-section/total C-section. The significance limit of *p* < 0.05 was defined for all tests.

**Results:**

The C-section rates were 50.8% at the HA and 42.3% at the HB, with 1.20 RR (95%CI = 1.13–1.28) at the HA. The highest rates were observed in Robson groups G5, G1, and G2. At the HA, G1 had a 21.5% C-section rate, which was greater than at the HB (13.8%; *p* < 0.05); the cesarean rates for groups G2 and G5 were higher at the HB (respectively, 18.6 and 38.1%) than at the HA (14.8 and 32.5%, respectively; *p* < 0.05).

**Conclusion:**

These results point out specific goals to be achieved in order to reduce abusive cesarean rates in both A and B hospitals, especially in the primigravida and in those with previous C-section.

## Introduction

The global increase in cesarean rates is real. High cesarean rates are becoming a public health problem and a reason for debate about the potential maternal and perinatal risks and the risks related to the costs of and inequalities in access to obstetric care [1,2]. Based on the rates of nations with low maternal and perinatal mortality, the WHO recommended in 1985 that the rate of cesarean births should not exceed 15% [3]. Since then, this rate has become a global goal.

According to a study conducted in Latin America in 1999, seven of 19 countries have cesarean rates below 15%: Haiti, Guatemala, Bolivia, Peru, Paraguay, Honduras and El Salvador. However, in the remaining 12 countries, the rates ranged from 16.8% to 40%; among these countries, Chile (41.0%), Brazil (27.1%), Dominican Republic (25.9%) and Argentina (25.4%) had the highest rates, while Colombia (16.8%), Panama (18.2%) and Ecuador (18.5%) had the lowest [4,5]. In Brazil, the percentage of births by C-section in 2001 was 53.88%, corresponding to 58.94% in the Midwest region and 52.77% in the Federal District [6].

The proposal and implementation of measures to reduce cesarean rates present large challenges and require critical study to identify the highest-risk mothers. In 2001, Robson proposed a simple, clinically relevant, reproducible and reliable classification system for cesareans. This classification system is the monitoring and audit tool that best meets local and international needs by including data commonly recorded at institutions providing different levels of care [7–9]. This instrument has been used in the United States and in other countries where there is interest in reducing the cesarean rates and improving obstetric care [5]. The protocol is based on maternal, pregnancy and delivery characteristics–parity, type of previous delivery (vaginal birth or C-section), gestation type (singleton or multiple), start of labor (induced or spontaneous) and gestational age; various characteristics form the 10 fully inclusive and mutually exclusive Robson groups [7]. In Brazil, this instrument has already been used to monitor obstetric practices hospital in a large maternity, with good results [10].

The WHO, in its statement of April 10, 2015, proposed that the Robson classification of C-sections be used as a global standard to assess, monitor and compare cesarean rates over time at the same hospital or among different hospitals in the same region or country [11]. The high cesarean rates registered in the capital of Brazil [12], which are far beyond the 15% recommended by the WHO [3], and the need for strategies to reduce these rates justify the design of the present study. The objective of this study was to evaluate the cesarean rates and the groups with the highest risk of C-section at the Hospital A (HA) and at Hospital B (HB), which are the reference hospitals for obstetric care in the SES-DF/Brazil, using the Robson classification for C-sections [7], and identify goals to reduce these rates.

## Materials and method

### Setting, design and source of data

The Federal District (DF, for its abbreviation in Portuguese) of Brazil comprises the city of Brasilia, the capital of Brazil, and other territories, and is divided into 31 Administrative Regions (AR) [13]. The network of the DF Secretary of Health (SES-DF, for its abbreviation in Portuguese) includes two public hospitals–here denominated HA and HB—which are reference maternity hospitals for 15 of the 31 ARs of the DF [14]. The monthly per capita income of the population in the Federal District is 2 to 3 minimum wages, and 90.6% of this population uses the Brazilian Unified Health System (SUS), defined by law as a universal, tax-funded, and national health system [15,16]. Thus, the maternity wards of these two hospitals represent the state of affairs for obstetric care within the SES-DF/Brazil, which is information of interest for improving the quality of obstetric care mainly for the middle and low-income population.

This is a cross-sectional study that included all births, vaginal or C-section, performed from January 1, 2013, to December 31, 2013, at two public hospitals in the SES-DF/Brazil network: the HA and the HB.

**Fig 1:**
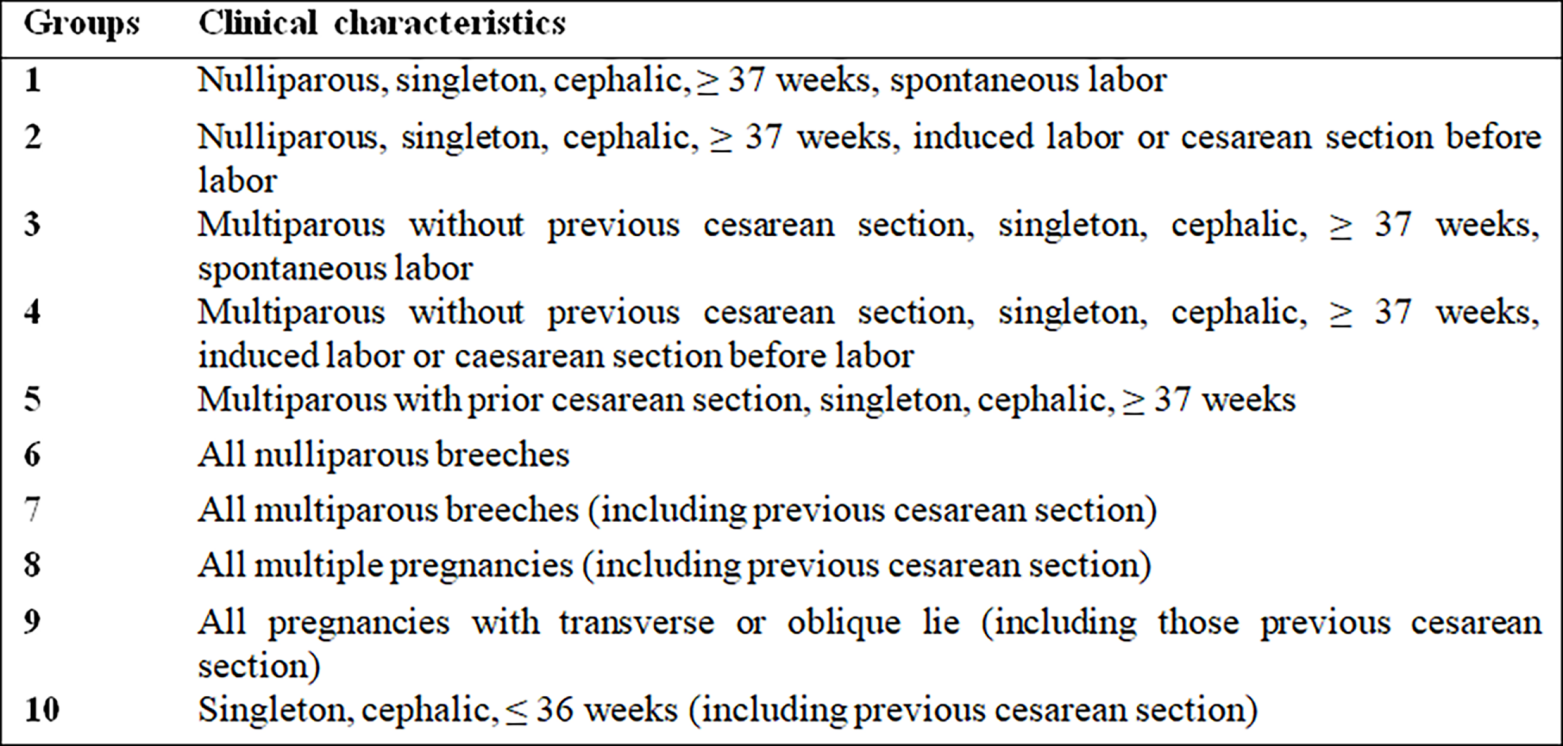
Cesarean section groups according to the Robson classification, 2001 [7].

During the study period, 6579 births occurred at the two hospitals, 4659 at the HA and 1920 at the HB, thus defining a convenience sample. The data were collected prospectively from the Intersystems track Care^TM^ electronic medical records system, which is available throughout the health network of SES-DF/Brazil. The maternal, pregnancy and birth characteristics were identified, and the births were distributed into the 10 Robson groups [7] (Fig 1).

The study was approved by the Research Ethics Committee of the Health Sciences Education and Research Foundation (FEPECS/SES, for its abbreviation in Portuguese), decision no. 127318.

### Definition of variables

The variables were: (I) Gestational age (GA), in full weeks at the time of the birth: calculated by the date of the last menstrual period (LMP) and/or ultrasound (USG) performed up to the 20^th^ week of gestation. (II) Type of gestation: defined as singleton (the presence of a single fetus) or multiple (more than one fetus). (III) Fetal presentation: cephalic or breech, with the fetus positioned longitudinally, and abnormal lie, with the fetus positioned transversely or obliquely. (IV) Parity: nulliparous (women who have never given birth) or multiparous (at least one previous birth). (V) Previous C-section: presence of a uterine scar from a C-section for a previous gestation. (VI) Onset of labor: spontaneous, induced by oxytocic agents, or absent, when the C-section was performed before the onset of labor.

### Statistical analysis

The statistical analysis was performed using SAS for Windows v.9.3 Software. The C-section rates in each group and its respective contribution to the total hospital C-sections was compared between HA and HB. To this, was used the proportion difference test (similar to chi-square test), with RR and 95% CI, and the logistic regression analysis (OR; 95% CI) among the groups with higher C-section/total C-section. The significance limit of p < 0.05 was defined for all tests.

## Results

During the period studied, 6579 births occurred; 3398 (51.6%) were vaginal births, and 3181 (48.4%) were C-sections. The Table 1 show the baseline characteristics of mothers at the hospital HA and hospital HB. The HA was responsible for 4659 (70.8%) of these births, of which 2290 (49.2%) were vaginal and 2369 (50.8%) were C-sections. At the HB, there were 1920 (29.2%) births, of which 1108 (57.7%) were vaginal and 812 (42.3%) were C-sections. The difference in cesarean rates between these two hospitals was statistically significant (*p* < 0.0001), with a greater frequency and risk at the HA (RR = 1.2; CI95% = 1.13–1.28) (Table 2).

**Table 1:** Baseline characteristics of mothers at the HA and HB hospitals / SES-DF/Brazil, during the study period.

	HA		HB		
	N	%	N	%	*p*
Maternal age (years)					
≤ 19	787	16,9	300	15,6	0.1471
20–34	3255	69,9	1325	69	0.1503
≥ 35	616	13,2	295	15,4	**0.0421**
Parity					
Zero	2172	46,6	810	42,2	**<0.0001**
≥ 1	2486	53,4	1110	57,8	
Onset of labor					
Spontaneous	3390	72,8	1301	67,8	**<0.0001**
Induced	395	8,5	274	14,3	**<0.0001**
C-section before labor	873	18,7	345	18	0.4845
Previous C-section					
Zero	3628	77,9	1498	78	0.9316
≥1	1030	22,1	422	22	
Type of gestation					
Single	4580	98,3	1891	98,5	0.7105
Multiple	78	1,7	29	1,5	
Type of fetal presentation					
Cephalic	4456	95,7	1853	96,5	0.1314
Breech	182	3,9	60	3,1	0.1442
transverse or oblique	20	0,4	7	0,4	0.8717
Gestational age (weeks)					
< 37	569	12,2	161	8,4	**<0.0001**
≥ 37	4089	87,8	1759	91,6	

Chi-square test. Significant statistical differences are highlighted in bold (*p*< 0.05)

**Table 2:** Distribution of births, vaginal and C-section at the HA and HB hospitals of the SES-DF/Brazil during the study period.

	Vaginal birth		C-section		*Total*	
	N	%	N	%	N	%
HA	2290	49.2	2369	50.8	4659	70.8
HB	1108	57.7	812	42.3	1920	29.2
*Total*	3398	51.6	3181	48.4	6579	100.0

C-section: HA *vs*. HB Chi-square test (*p*< 0.0001); C-section: HA *vs*. HB (RR = 1.20; IC95%1.13;1.28)

Relative to the number of births, the groups G1, G3 and G5 represented those the most frequency. Relative to the percentages of C-sections, the groups G1, G2, G3, G4 and G5 showed a significative difference (*p* < 0.05), always higher in the HA (36.2 *vs*. 22.3% in G1, 82.6 *vs*. 70.6% in G2, 14.1 *vs*. 7.2% in G3, 62 *vs*. 46.7% in G4 and 87 *vs*. 82% in G5). The relation C-section/total C-section was more prevalent and statistically different in groups G1, G2 and G5 in both HA and HB. In the groups G5 (38.1 *vs*. 32.5%; *p* = 0.004) and G2 (18.6 *vs*. 14.8%; *p* = 0.011), the highest rates were observed at the HB. In the group G1, the highest rates were observed at the HA (21.5 *vs*. 13.8%; *p* < 0.0001). The cesarean rates of the groups G1, G2 and G5 represented the major contributions to the total C-sections, with a significant difference in G1. In this group, C-section rates were more elevated in the HA (*p* < 0.0001) (Table 3).

**Table 3:** Distribution of births and C-section at the HA and HB hospitals of the SES-DF/Brazil during the study period according to the 10 Robson groups [7].

Robson groups	Birth / groups N (%)			% C-section / groups N (%)			C-section / total C-section N (%)			C-section / total birth N (%)		
	HA	HB	*p*	HA	HB	*P*	HA	HB	*p*	HA	HB	*p*
1	1409/4659 (30.2)	503/1920 (26.2)	**0.0010**	510/1409 (36.2)	112/503 (22.3)	**< .0001**	510/2369 (21.5)	112/812 (13.8)	**< .0001**	510/4659 (11.0)	112/1920 (5.8)	**< .0001**
2	425/4659 (9.1)	214/1920 (11.1)	**0.0119**	351/425 (82.6)	151/214 (70.6)	**0.0005**	351/2369 (14.8)	151/812 (18.6)	**0.0110**	351/4659 (7.5)	151/1920 (7.9)	0.6460
3	981/4659 (21.0)	471/1920 (24.5)	**0.0119**	138/981 (14.1)	34/471 (7.2)	**0.0003**	138/2369 (5.8)	34/812 (4.2)	0.0988	138/4659 (3.0)	34/1920 (1.8)	**0.0093**
4	266/4659 (5.7)	137/1920 (7.1)	**0.0286**	165/266 (62.0)	64/137 (46,7)	**0.0034**	165/2369 (7.0)	64/812 (7.9)	0.3834	165/4659 (3.5)	64/1920 (3.3)	0.6754
5	884/4659 (18.9)	377/1920 (19.6)	0.5355	769/884 (87.0)	309/377 (82.0)	**0.0178**	769/2369 (32.5)	309/812 (38.1)	**0.0042**	769/4659 (16.5)	309/1920 (16.1)	0.6591
6	85/4659 (1.8)	25/1920 (1.3)	0.1349	85/85 (100.0)	25/25 (100.0)	—	85/2369 (3.6)	25/812 (3.1)	0.4605	85/4659 (1.8)	25/1920 (1.3)	0.1214
7	66/4659 (1.4)	27/1920 (1.4)	0.9742	66/66 (100.0)	26/27 (96.3)	0.5889	66/2369 (2.8)	26/812 (3.25)	0.7695	66/4659 (1.4)	25/1920 (1.4)	0.6037
8	76/4659 (1.6)	27/1920 (1.4)	0.5043	61/76 (80.3)	23/27 (85.2)	0.9993	61/2369 (2.6)	23/812 (2.8)	0.9447	61/4659 (1.3)	23/1920 (1.2)	0.4856
9	16/4659 (0.3)	5/1920 (0.3)	0.5886	16/16 (100.0)	5/5 (100.0)	0.9994	16/2369 (0.7)	5/812 (0.6)	0.9568	16/4659 (0.3)	5/1920 (0.3)	0.6808
10	451/4659 (9.7)	134/1920 (7.0)	**0.0005**	208/451 (46.1)	63/134 (47.0)	0.9994	208/2369 (8.8)	63/812 (7.8)	0.5667	208/4659 (4.5)	63/1920 (3.3)	0.0613
Total	4659/4659 (100.0)	1920/1920 (100.0)		2369/4659 (50.8)	812/1920(42.3)		2369/2369 (100.0)	812/812 (100.0)		2369/4659 (50.8)	812/1920 (42.3)	

Chi-square test. Significant statistical differences are highlighted in bold (*p*<0.05).

The risk analysis for the occurrence of C-section in groups with the highest rates indicated that: compared to the G1, groups G2 and G5 represents a 45–50% decrease in the risk of C-section; group G1 corresponds to an increase of about 1.8 to 2.0 times in the risk for C-section in relation to the G2 and G5; the risk for C-section was not statistically different between the groups G1 and G5 (table 4).

**Table 4:** Logistic regression analysis relative to C-section risk in Robson groups G1, G2 and G5 at the HA and HB hospitals / SES-DF/Brazil, during the study period.

Models tested	OR	IC95%
G2 *vs*. G1	**0.51**	0.39–0.68
G5 *vs*. G1	**0.55**	0.43–0.70
G1 *vs*. G2	**1.96**	1.50–2.60
G5 *vs*. G2	1.07	0.85–1.35
G1 *vs*. G5	**1.83**	1.43–2.33
G2 *vs*. G5	0.93	0.74–1.18

Significative statistical results are highlighted in bold (*p*< 0.05)

## Discussion

The results of this study showed high rates of C-sections at the both hospital HA (50.8%) and the hospital HB (42.3%) in the study period. The occurrence of C-section in the 10 Robson groups [7] was also high; group G3 was the only exception. These rates, whether the totals at the hospital level or broken down according to Robson group, are far higher than the 15% recommended by the WHO [3] and the average of 38.5% cesarean rates registered in the SES-DF/Brazil for this same period [17].

In this study, groups G5, G1 and G2 contributed most to the hospital cesarean rates; they were responsible for 68.8% of the cesareans performed at the HA and 70.5% of those performed at the HB. Corroborating our results, the study by Brennan et al. [18] identified the contribution of these groups (G5, G1 and G2) to more than 50% of cesareans performed at various institutions from different countries and continents.

The group G1 is defined by nulliparous term pregnancies with spontaneous labor, the group G2 is composed of nulliparous pregnancies with induced labor or C-section before the onset of labor and group G5 comprises multiparous pregnancies with previous C-section. The percentage of births and C-sections/group and the contribution of this percentage to the hospital rate of C-sections identifies these three groups as a priority for specific goals: (I) to accept birth as a natural and physiological event; (II) to avoid the first C-section (intrapartum or before the onset of labor), and (III) to break away from the paradigm "once a cesarean, always a cesarean". Waiting for the natural evolution of labor without inducing or manipulating the uterine contractions and not scheduling repeat C-sections or those without medical indication could be important strategies for reducing the cesarean rates of these hospitals.

In Brazil, it is still common practice to induce or stimulate uterine contractions of labor with oxytocin, especially in private medical practice [19–22]. However, when indicated, the adoption of strict protocols and careful evaluations of favorable conditions for induction is recommended, along with ripening the unfavorable cervix in advance when necessary [23–27]. Admitting women who are not in active labor presents a 15.8% probability and a 2.5 times higher risk of C-section [28]. The repeat C-section is a facilitating factor for new cesareans, and a trial of labor in women with previous C-section can prevent unnecessary cesareans in these cases [29–32]. With care and observation, a trial of labor can be the strategy of choice for pregnant women with prior cesarean scar and adequate fetal weight to avoid up to 80.0% new cesareans without maternal or fetal risk [33]. Similarly, routinely using a partogram to monitor labor [34,35] and obtaining a second opinion regarding the indication for C-section [36] could support a reduction in C-sections, particularly in intrapartum cases and in women with prior vaginal birth experience [37–39].

Our results suggest that the induction of labor is not to be common practice in the two hospitals, and must have influenced the percentage of C-sections in group G2 where the rates of C-section were 34,7% in women who induced labor *vs*. 65,3% C-section in women who are not in active labor. Partogram use is not documented in the electronic system used to record births in the SES-DF/Brazil network; therefore, no data on partogram use was available for the two studied hospitals. Thus, the lack of a partogram to monitor labor could have influenced the cesarean rates of these hospitals and especially the indications for intrapartum cesareans in groups G1 and G2. On the other hand, the repeat cesareans must have contributed to the high rates observed in group G5. This indicates the need to institute a trial of labor for women in this group to reduce the group’s 80.0% cesarean rate. Despite this, considering that the risk of C-section, being to the group G1 corresponds to an increase of about 1.8 to 2.0 times in the risk for C-section in relation to the G2 and G5.

Group G3, which is characterized by multiparous term pregnancies without cesarean scar and spontaneous labor, represented approximately 20% of the births evaluated and less than 15% of the cesareans; thus, it had a minimal contribution to the total cesarean and birth rates of the two hospitals. Compared with other groups, this group would not be prioritized for the implementation of C-section prevention strategies. However, considering that vaginal births with few to no interventions and at most 2% C-section births [40] would be the natural evolution expected for this group, the cesarean rates of 14.2% at the HA and 7.1% at the HB are beyond what is acceptable.

For group G4, which had the same characteristics as G3 except that they had induced labor or had a C-section before the onset of labor, the cesarean rates were 62.0% at the HA and 46.7% at the HB. Despite this group’s low representativeness in terms of the number of births and its contribution to the hospitals’ cesarean rates, the results of this group must be considered. Avoiding the induction of labor and the first C-section would be specific goals for this group. To do this, the judicious assessment of indications and cervical conditions in cases of induction of uterine contractions [23–27], the admission of women in active labor [28], the routine use of a partogram [34,35] and a second opinion on indications for C-section [36] would be specific strategies for this group.

Groups G6 to G9 had cesarean rates of 80 to 100%. Despite the alarming rates, there were fewer births in these groups, and their contributions to the hospital cesarean rates did not exceed 2%. However, it is concerning that at the studied hospitals, virtually all mothers belonging to these groups underwent a C-section. The common characteristic of groups G6, G7 and G9 is the presence of anomalous presentations, and group G8 comprises multiple pregnancies regardless of prior C-section.

Although the evidence indicates that C-section is the most appropriate birth method in breech presentations [23], the practice of external cephalic version in term pregnancies [41,42] should be implemented in these services as a prevention measure to reduce the cesarean rates in groups G6 and G7 (fetus in breech presentation) and in group G9 (fetus in transverse of oblique presentation).

Twin pregnancies have a low rate of occurrence. In most cases, they are dichorionic and diamniotic, and the presentation of the first fetus is cephalic. The indication for the method of birth is, preferably, obstetric and driven by the presentation of the first twin [43–46]. However, with the increasing trend in assisted reproductive technologies [47], multiple pregnancies are likely to become more frequent and further increase the cesarean rates of 80 to 85% observed in group G8. Thus, the recommendations regarding the method of birth for twin pregnancies [46] and the practice of external cephalic version for term pregnancies [41,42] should be the strategies of choice at the HA and the HB. Because of the difficulties inherent to twin births and the need for skilled professionals trained to deliver twins, it would also interesting for hospitals to invest in the training of a specific team for this type of birth.

Considering the frequency of births in group G10 (7 to 10%) and its contribution of less than 5% of the total number of cesareans in the evaluated hospitals, this group would not be a priority for interventions to reduce the number of cesareans. This group is defined by premature births; in our study, 50% of these births were C-section. Maternal complications are responsible for 45.9% of pre-term births; in particular, arterial hypertension in all its forms is associated with 70.7% of pre-term births and 76.7% of C-sections [48–50]. Nevertheless, the C-section is not always the birth method of choice in cases of pre-term labor [49], and the critical assessment of its indication should reduce the cesarean rates observed in our study.

The results of this study confirmed high cesarean rates at the two hospitals of the SES-DF/Brazil. Except for women in the group G3, all other women admitted to the HA or the HB have a potential risk of undergoing a C-section. In practice, only multiparous women at term, without prior C-section, and who had the chance to wait for spontaneous labor had no risk of C-section. Belonging to any other of the nine Robson groups [7] implies a high risk of C-section, which is alarming.

This study has some limitations that should be considered. The data collecting from an electronic medical record system, without access to the attendant professional, did not allow a regression analysis adjusted for possible confounding variables, especially comorbidities. The reduced number of subjects in some Robson’s groups may have limited the sample size and influenced the statistical power of the results. Despite this, our results indicate the need for specific interventions to reduce the alarming C-sections rates in groups G5, G1 and G2 of both A and B hospitals in SES-DF/Brazil.

## Conclusions

In this study, the C-section rates were 50.8% at the HA and 42.3% at the HB, with 1.20 RR (95%CI = 1.13–1.28) at the HA. The groups G5, G1 and G2 were the major contributes to these elevated rates. These results point out goals to be achieved in order to reduce abusive cesarean rates in both A and B hospitals, especially in the primigravida and in those with previous C-section. Among them, avoid the first C-section and wait for the natural spontaneous labor; break the paradigm "once cesarean always cesarean" and include the VBAC in the care protocol. Moreover, institute the partogram and the C-section second opinion should support these goals.

## Supporting information

S1 File Dataset. (XLSX)
